# Supplementary material for: Effects of Different Physical Activity Levels during a Single Day on Energy Intake, Appetite, and Energy Balance: A Preliminary Study
Source: Nutrients. 2019 Mar 23;11(3):690. doi: 10.3390/nu11030690 (PMC6471929; doi:10.3390/nu11030690)
Supplement: Supplementary file 1 [file nutrients-11-00690-s001.pdf]

Table S1. Buffet items at dinner.
Participants freely ate the food items until they were satisfied, and the duration of buffet time was from 19:30 to 23:00.

Table S1. Buffet items at dinner (19:30-23:00)
•Beef pastrami
•Curry
•Fried chicken
•Chocolate cookie
•Butter cookie
•Rice crackers
•Cheese
•Fish sausage
•Steamed Chicken
•Bean-starch vermicelli salad
•Coleslaw
•Rice
•Boiled eggs
•Dumplings
•A peace of cake（Baumkuchen）
